# Facile Fabrication of Bi_2_WO_6_/Ag_2_S Heterostructure with Enhanced Visible-Light-Driven Photocatalytic Performances

**DOI:** 10.1186/s11671-016-1319-7

**Published:** 2016-03-08

**Authors:** Rongfeng Tang, Huaifen Su, Yuanwei Sun, Xianxi Zhang, Lei Li, Caihua Liu, Bingquan Wang, Suyuan Zeng, Dezhi Sun

**Affiliations:** Shandong Provincial Key Laboratory of Chemical Energy Storage and Novel Cell Technology, Department of Chemistry and Chemical Engineering, Liaocheng University, Liaocheng, 252059 China

**Keywords:** Bi_2_WO_6_/Ag_2_S heterostructures, Enhanced photocatalytic activity, Visible light driven, pH dependent, Photocatalytic mechanism

## Abstract

In this report, a novel photocatalyst based on Bi_2_WO_6_/Ag_2_S heterostructures was prepared by a 3-mercaptopropionic acid (MPA)-assisted route at room temperature. Compared to bare Bi_2_WO_6_ and Ag_2_S nanoparticles, the as-formed Bi_2_WO_6_/Ag_2_S heterostructures exhibit enhanced photocatalytic activity for the degradation of rhodamine B (Rh B) under visible-light irradiation. This kind of enhancement in the photocatalytic activity is considered to be the synergistic effects of both the effective electron-hole separation and expansion of the light-absorption range. The pH of the solution is of vital importance to the photocatalytic activity of the as-formed Bi_2_WO_6_/Ag_2_S heterostructures. Under low pH value, the photosensitization process is suppressed, while under higher pH value, the photosensitization process is favored. The mechanism of the photocatalytic process was proposed by the active-species-trapping experiments, indicating that the photogenerated holes (h^+^) play a crucial role in the degradation of Rh B under visible light. The enhanced photocatalytic performance of this heterostructure makes it a promising material for the treatment of dye-containing wastewater.

**Electronic supplementary material:**

The online version of this article (doi:10.1186/s11671-016-1319-7) contains supplementary material, which is available to authorized users.

## Background

With the widespread application of visible-light-driven photocatalysts, Bi_2_WO_6_ has attracted more and more attention because of its unique crystal structure and physicochemical properties [1–3]. As the simplest member of the Aurivillius oxide family, Bi_2_WO_6_ exhibits good photocatalytic performance under visible-light irradiation. Therefore, many efforts have been devoted for the preparation of Bi_2_WO_6_-based photocatalysts, such as the solid-state method [4], microwave-solvothermal method [5], ultrasonic synthetic method [6], and hydrothermal reactions [7]. However, Bi_2_WO_6_ can only respond to visible light with the wavelength shorter than 450 nm [8], which accounts for only a small part of the solar light. Meanwhile, the rapid recombination of the photo-induced electron-hole pairs also greatly decreases the photocatalytic activity of Bi_2_WO_6_, which prevents it from further large-scale applications [9]. To broaden the light-absorption range and promote the separation of photogenerated carriers of Bi_2_WO_6_, two main methods are employed. The first one is based on the element doping of Bi_2_WO_6_ (such as B, Gd, Ag, N, Ce, and F codoping) [10–14]. The other method is based on the formation of heterostructures between Bi_2_WO_6_ and other kind of materials, such as g-C_3_N_4_ [15], C60 [16], graphene [17], metals [18, 19], and various semiconductors.

The combination of Bi_2_WO_6_ with other semiconductors has been proved to be an effective method for the preparation of the photocatalysts with enhanced photocatalytic performances. On the one hand, the coupling of Bi_2_WO_6_ with other semiconductors will broaden the light-absorption range via the formation of intermediate energy levels. On the other hand, the recombination rate of photo-induced charge carriers will be decreased because of the charge transfer on the interfaces of heterostructures. As a result, a variety of heterostructures based on Bi_2_WO_6_ have been successfully prepared, which exhibit enhanced photocatalytic activities under visible light. For instance, Yang and coworkers reported the preparation of a BiOCl-Bi_2_WO_6_ heterojunction with a chemically bonded interface. The decomposition rate constant for rhodamine B is about 2 times faster than that for pure BiOCl (0.029 min^−1^) and 1.5 times faster than that for Bi_2_WO_6_ (0.041 min^−1^) [20]. Zhang and coworkers have also succeeded in the preparation of a novel Bi_2_S_3_/Bi_2_WO_6_ composite photocatalyst using hydrothermal method. The apparent rate constant is calculated to be 0.0062 min^−1^ for the Bi_2_S_3_/Bi_2_WO_6_ composite, which is 6.2 times higher than the corresponding value of bare Bi_2_WO_6_ (0.001 min^−1^) [8]. Other kinds of heterostructures such as Bi_2_WO_6_/α-Fe_2_O_3_ [21], Bi_2_WO_6_/TiO_2_ [22], Bi_2_WO_6_/BiOBr [23], Bi_2_WO_6_/BiIO_4_ [24], and Bi_2_WO_6_/BiVO_4_ [25] have also been successfully synthesized, all of which exhibit enhanced photocatalytic activities as compared to bare Bi_2_WO_6_. However, developing new heterostructures based on Bi_2_WO_6_ is still a big challenge for the chemists, especially by a simple and economic method.

As a semiconductor with narrow bandgap (1.0 eV), Ag_2_S has been widely used in various fields such as photoconductors, photovoltaic cells, IR detectors [26, 27], photography [28], and luminescent devices [29]. Because of its narrow bandgap, Ag_2_S can absorb light with the wavelength lower than 1000 nm, which covers the whole visible-light region. Meanwhile, the conduction band (CB) and valence band (VB) position of Ag_2_S is higher than the corresponding values of Bi_2_WO_6_, which can form the type-II heterostructures when coupling with Bi_2_WO_6_. These two fascinating characteristics make the Bi_2_WO_6_/Ag_2_S heterostructure a good candidate for the photodegradation of organic dyes. However, few reports are concerned on the fabrication and photocatalytic activity of Bi_2_WO_6_/Ag_2_S heterostructure. In this report, the Bi_2_WO_6_/Ag_2_S heterostructures were successfully prepared by a surface functionalization method using 3-mercaptopropionic acid (MPA) as the surface-functionalizing agent. The as-formed Bi_2_WO_6_/Ag_2_S heterostructures exhibit enhanced photocatalytic activity as compared to bare Bi_2_WO_6_ and Ag_2_S. Accordingly, a rational model is proposed to illustrate the key roles of Ag_2_S in the photocatalytic process and the corresponding photocatalytic mechanism of the as-formed heterostructure is also proposed.

## Methods

All the reagents are commercially available and used without further treatments.

### Synthesis of Flower-Like Bi_2_WO_6_

The flower-like Bi_2_WO_6_ were synthesized by a hydrothermal method as we have previously reported [30]. In a typical process, 2 mmol Bi(NO_3_)_3_·5H_2_O and 1 mmol Na_2_WO_4_·2H_2_O were added to 22.5 mL of deionized water under magnetic stirring, respectively. Then, the two solutions were mixed and stirred for another 30 min. The resulting white suspension was then transferred into a 50-mL Teflon-lined autoclave and heated at 200 °C for 12 h. After cooling to room temperature naturally, the precipitates were collected by centrifugation, washed with deionized water and ethanol, and then dried at 60 °C for 6 h in vacuum.

### Synthesis of Bi_2_WO_6_/Ag_2_S Heterostructures

Bi_2_WO_6_/Ag_2_S heterostructures were prepared by a surface functionalization route which employs MPA as the surface-functionalizing agent [31]. In a typical process, 1 g of Bi_2_WO_6_ was dispersed in 40 mL of deionized water to form a slurry under magnetic stirring. Then, 20 μL of MPA was added into the above suspension, followed by vigorous stirring for 4 h to ensure the complete surface functionalization of Bi_2_WO_6_. In the next step, 0.05 g of AgNO_3_ was added to the above reaction mixture and the suspension was stirred for another 2 h at room temperature. At last, 0.03 g of Na_2_S·9H_2_O was added dropwise to the abovementioned system. The resulting suspensions were stirred at room temperature for another 1 h. The molar ratio between elements Ag and S was 2:1. Finally, the product was separated by centrifugation, washed with ethanol and water for several times, and dried under vacuum at 60 °C to obtain the Bi_2_WO_6_/Ag_2_S heterostructures. The resulting powder was collected for further characterization.

### Synthesis of Ag_2_S Nanoparticles

For comparison purpose, Ag_2_S nanoparticles were also synthesized by a simple precipitation method. In a typical process, 1.0 g of AgNO_3_ was dissolved in 40 mL of deionized water to form a transparent solution. Then, 0.71 g of Na_2_S·9H_2_O was added to the above solution and stirred for another 1 h at room temperature. The resulting products were separated by centrifugation, washed with deionized water and absolute alcohol for 3 times, and then dried at 60 °C for 12 h in vacuum.

### Sample Characterizations

X-ray diffraction (XRD) patterns were monitored by a Philips X’Pert Pro Super diffractometer using Cu Kα radiation (*λ* = 1.5416 Å). The scanning rate of 0.05°s^−1^ was applied to record the patterns in the 2*θ* range of 10°–70°. The scanning electron microscope (SEM) characterizations were performed on the S-4800 (Hitachi) field emission scanning electron microscope (FESEM) equipped with a GENESIS4000 energy-dispersive X-ray spectroscope. The transmission electron microscope (TEM) analyses were performed using a Hitachi H-7650 transmission microscope at an accelerating voltage of 100 kV, and the high-resolution transmission electron microscopy (HRTEM) images were obtained on a JEOL-2010 TEM at an acceleration voltage of 200 kV. The Brunauer-Emmett-Teller (BET) tests were determined via a Quantachrome autosorb IQ-C nitrogen-adsorption apparatus. All the as-prepared samples were degassed at 150 °C for 4 h prior to nitrogen-adsorption measurements. X-ray photoelectron spectroscopy (XPS) analysis was performed on Thermo ESCALAB 250 system with a monochromatic Al Kα source with 1486.6 eV of energy and 150 W of power. The light-absorption properties were measured using a UV-vis diffuse reflectance spectrophotometer (DRS) (Shimadzu, UV-2550) by using BaSO_4_ as the background at room temperature and were converted from reflection to absorbance by the Kubelka-Munk method. The photoluminescence (PL) spectra of the samples are recorded with the F-7000 FL spectrophotometer.

### Photocatalytic Experiments

The photocatalytic activities of the photocatalysts were evaluated by the degradation of rhodamine B (Rh B) using a 500-W Xe lamp with a 400-nm cutoff filter. The working distance from the Xe lamp to the beaker is 20 cm. In this process, 80 mg of the photocatalyst was added into 80 mL Rh B solution (10^−5^ mol L^−1^). Temperature of the beaker containing the dispersion of Rh B and the samples was maintained at 20 °C by using circulating water during the whole process. Prior to irradiation, the mixture was magnetically stirred in the dark for 30 min to ensure the adsorption/desorption equilibrium between the photocatalyst and Rh B. Then, light was turned on, and optical power is maintained at 25.8 mW cm^2^. At given time intervals, 6 mL of the suspension was taken out and centrifuged at 9000 rpm to remove the residual photocatalyst powders for analysis. The clear solution was analyzed through UV-vis spectrophotometer (Agilent Cary 5000E) by recording the variations of the absorption band at 553 nm.

### Active-Species-Trapping Experiments

For the detection of active species generated in the photocatalytic reaction, active-species-trapping experiments were carried out. In this process, various kinds of scavengers such as benzoquinone (BQ) (a quencher of O_2_^·−^), ammonium oxalate (AO) (a quencher of h^+^), AgNO_3_ (a quencher of *e*^−^), and t-BuOH (a quencher of ·OH) were employed. And the amount of scavenger was fixed to be 10 mM except BQ, which was 1 mM. The whole process was similar to the photocatalytic experiments mentioned above.

### Photocurrent Measurements

For the preparation of the photoelectrode, the ITO (Indium tin oxide) glass was firstly cut into 3 cm (length) × 0.7 cm (width) slices, washed with water and 1 M NaOH solution several times before use. In the next step, 10 mg of the photocatalysts was dispersed in 1 mL of absolute ethanol by ultrasonication. Then, 10 μL of the resulting dispersion was drop-casted onto the ITO slice by a pipette and the area was fixed to be 0.7 cm^2^. The as-prepared photoelectrodes were dried in the air for further investigation. The photoelectrochemical characteristics were measured using a CHI 660B (Chen Hua Instruments, Shanghai, China) electrochemical working station with a standard three-electrode configuration under visible light provided by a 500-W Xe lamp. The as-prepared photoelectrode, Pt wire, and Ag/AgCl electrode were used as the working electrode, counter electrode, and reference electrode, respectively. During the electrochemical test, 0.1 M phosphate-buffered saline (pH = 7.4) was used as the electrolyte, and the optical power density on the ITO electrode was determined to be 70 mW cm^2^.

## Results and Discussion

### Characterizations of the Bi_2_WO_6_/Ag_2_S Heterostructures

**Fig. 1:**
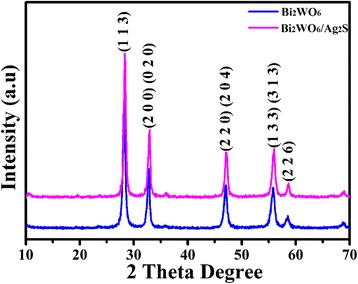
XRD patterns of the as-prepared Bi_2_WO_6_ and Bi_2_WO_6_/Ag_2_S

The phase purity of the as-prepared samples was investigated by the X-ray diffraction (XRD) method, and the corresponding result is shown in Fig. 1. All the diffraction peaks on Fig. 1 can be indexed to be the orthorhombic-phase Bi_2_WO_6_ with cell constants of *a* = 0.5456 nm, *b* = 0.5434 nm, and *c* = 1.64 nm, which is consistent with the previous reports (JCPDS Card, No. 73-1126) [32]. The XRD pattern of the as-obtained Bi_2_WO_6_/Ag_2_S heterostructure is also shown in Fig. 1 (purple line). Compared with the XRD pattern of Bi_2_WO_6_, no obvious change can be observed. The absence of the peaks corresponding to Ag_2_S may result from the low content and poor crystallinity of Ag_2_S nanoparticles.

**Fig. 2:**
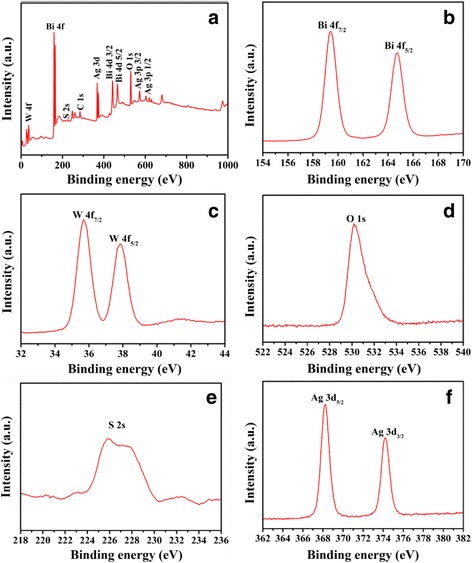
**a** The overall XPS spectrum of the as-prepared Bi_2_WO_6_/Ag_2_S heterostructures. XPS spectra of **b** Bi 4f, **c** W 4f, **d** O 1 s, **e** S 2 s, and **f** Ag 3d

To verify the existence as well as the chemical states of Ag_2_S in sample Bi_2_WO_6_/Ag_2_S, the as-prepared heterostructures were further investigated using X-ray photoelectron spectroscopy (XPS). The binding energies in the spectrum were calibrated using that of C 1s (284.62 eV). Figure 2a is the overall XPS spectrum of the Bi_2_WO_6_/Ag_2_S heterostructure, in which elements Bi, W, O, S, and Ag can be clearly detected. The peaks centering at 164.73 and 159.43 eV (Fig. 2b) can be designated to be the binding energies of Bi 4f_7/2_ and Bi 4f_5/2_. And the peaks centering at 37.83 and 35.68 eV (Fig. 2c) can be ascribed to W 4f_5/2_ and W 4f_7/2_. All the measured values are consistent with the previous reports [33, 34]. The peak with the binding energy of 226.4 eV (Fig. 2e) can be designated to be S 2S, while the peaks centering at 374.23 and 368.23 eV (Fig. 2f) can be ascribed to Ag 3d_3/2_ and Ag 3d_5/2_ [35]. Considering the binding energy of Ag 3d_3/2_ and Ag 3d_5/2_, the valence of Ag in the heterostructure can be identified to be +1. No peak corresponding to metallic Ag (0) was detected in the XPS spectrum, indicating that Ag mainly exists in the form of Ag_2_S. The atomic ratio of Bi:W:Ag:S is determined to be 2:1:1.19:1, which is much larger than the theoretical value (2:1:0.2:0.1). This phenomenon clearly indicates that the as-formed Ag_2_S are mainly dispersed on the surfaces of Bi_2_WO_6_ because the XPS is a surface analysis technique indeed.

**Fig. 3:**
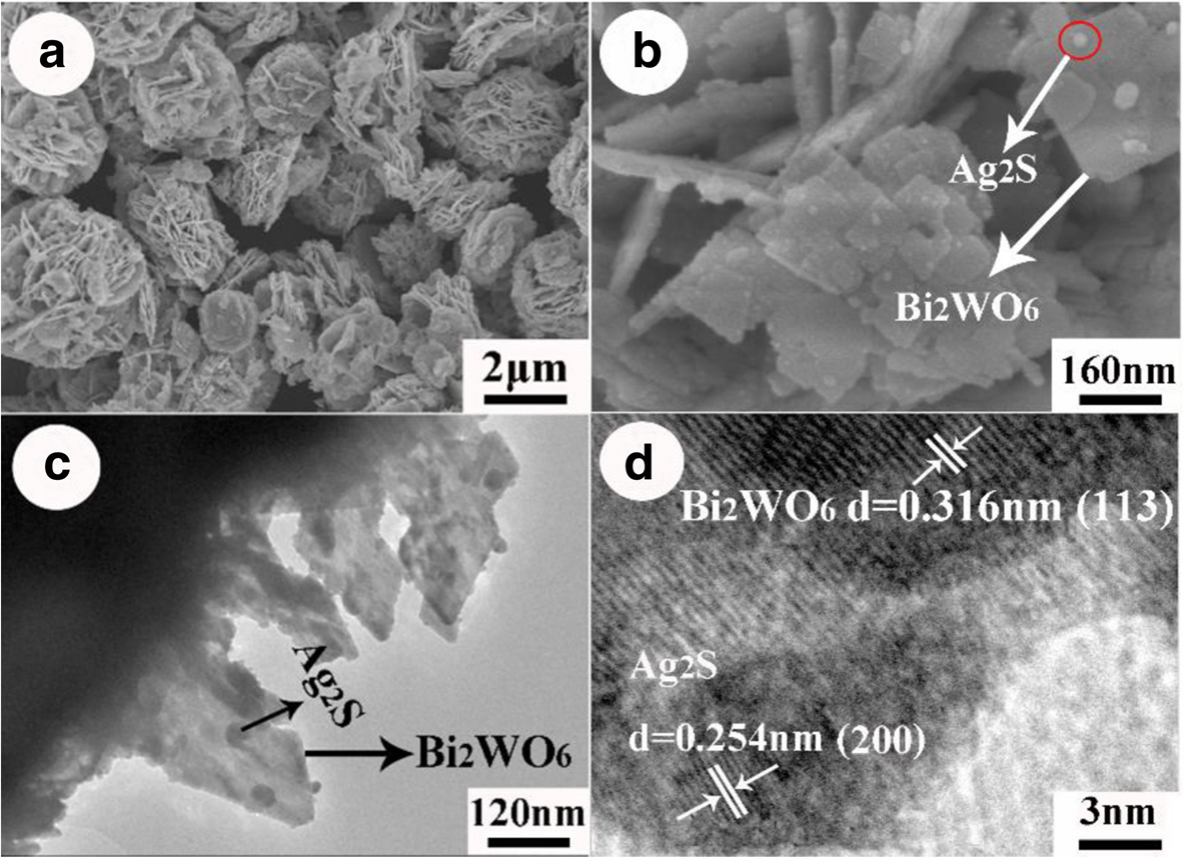
**a**, **b** SEM, **c** TEM, and **d** HRTEM images of the Bi_2_WO_6_/Ag_2_S heterostructure

The structure and morphology of the as-prepared Bi_2_WO_6_/Ag_2_S heterostructures were further investigated by SEM, TEM, and HRTEM. Figure 3a is the low-magnification SEM image of the as-prepared Bi_2_WO_6_/Ag_2_S heterostructures, which clearly indicates that the sample is composed of flower-like microstructures with diameter of about 2.0 μm. This result agrees well with the microstructure of bare Bi_2_WO_6_ in our previous report [30]. Figure 3b is the high-magnification SEM image of the as-prepared Bi_2_WO_6_/Ag_2_S heterostructure, suggesting the existence of small nanoparticles on the surfaces of these nanoplates. These nanoparticles, as investigated by the element mapping method, are determined to be Ag_2_S nanoparticles (Additional file 1: Figure S1). The corresponding ratios among elements Bi:W:Ag:S are determined to be 2:0.92:0.19:0.10. The existence of these small nanoparticles on the surfaces of Bi_2_WO_6_ nanoplates can be further verified by the TEM observation. These small nanoparticles, with diameters of about 10–20 nm, disperse uniformly on the nanoplates. According to the EDS analysis of the these small nanoparticles (Additional file 1: Figure S2), both elements S and Ag can be detected, which is consistent with the mapping result. To gain further insight into the detailed structure of sample Bi_2_WO_6_/Ag_2_S, HRTEM was employed. Figure 3d is the HRTEM image of sample Bi_2_WO_6_/Ag_2_S, which clearly indicates the formation of the Bi_2_WO_6_/Ag_2_S heterostructure. The lattice spacing taken on the tiny nanocrystals is measured to be about 0.254 nm, which is consistent with the “0 2 2” planes of Ag_2_S (JCPDS NO. 76-0134), indicating the existence of Ag_2_S nanoparticles. The typical lattice spacing, being determined to be 0.316 nm, is consistent with the “1 1 3” planes of Bi_2_WO_6_.

In general, the BET surface area of the photocatalyst can greatly affect its photocatalytic performance, and a high BET surface area is usually beneficial for the improvement of photocatalytic activity [36, 37]. The BET-specific surface areas of Bi_2_WO_6_ and Bi_2_WO_6_/Ag_2_S were determined by nitrogen-adsorption BET method. The specific surface areas (Additional file 1: Figure S3) of pure Bi_2_WO_6_ and Bi_2_WO_6_/Ag_2_S were determined to be about 13.6 and 11.0 m^2^ g^−1^, respectively. Compared with bare Bi_2_WO_6_, the surface area of the as-formed Bi_2_WO_6_/Ag_2_S heterostructure is a little smaller. The decrease in surface area may result from the surface coverage of Ag_2_S, and similar experimental results have also been observed in the plasmonic nanocomposite photocatalysts Ag/AgX-CNTs (X = Cl, Br, I) [38]. In this report, the specific surface area of Ag/AgCl-CNTs, Ag/AgBr-CNTs, and Ag/AgI-CNTs are 50.3, 20.8, and 18.4 m^2^ g^−1^, respectively, which are also a little smaller than the surface areas of CNTs (59.2 m^2^ g^−1^).

### Optical Property of the As-Formed Bi_2_WO_6_/Ag_2_S Heterostructure

**Fig. 4:**
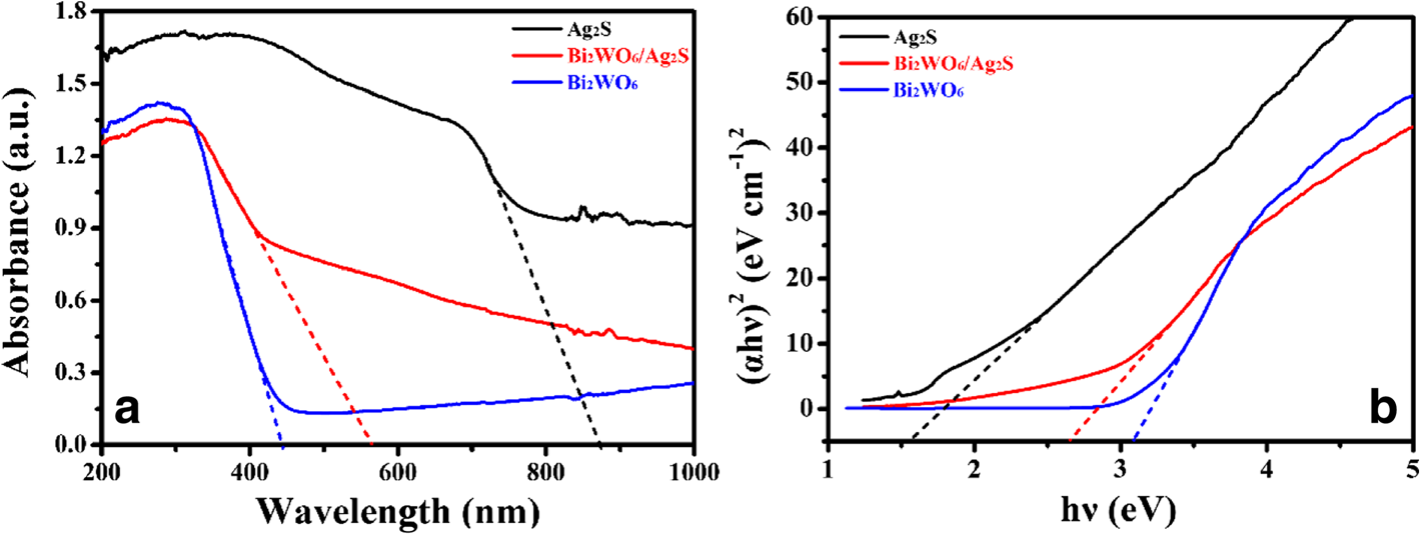
**a** UV-vis absorption spectra of Bi_2_WO_6_, Ag_2_S and Bi_2_WO_6_/Ag_2_S. **b** Kubelka-Munk plot for bandgap calculation of Bi_2_WO_6_, Ag_2_S, and Bi_2_WO_6_/Ag_2_S

Figure 4a is the UV-vis diffuse reflectance spectrum (DRS) of Bi_2_WO_6_, Ag_2_S and Bi_2_WO_6_/Ag_2_S heterostructures. Flower-like Bi_2_WO_6_ exhibits photo-absorption from UV light to visible light, and the absorption edge locates at ~450 nm. After surface modification with Ag_2_S, an obvious red shift in the absorption edge can be observed. The absorption edge of the Bi_2_WO_6_/Ag_2_S extends to ~550 nm because of the coupling of Ag_2_S nanoparticles with Bi_2_WO_6_ nanoplates. The optical bandgaps of a semiconductor can be calculated using the following equation [39]:

$$ {\left(\alpha hv\right)}^{\frac{1}{n}}=A\left(hv-{E}_{\mathrm{g}}\right) $$

where *α* is the absorption coefficient, *h* is the Planck constant, *ν* is the frequency of vibration, *A* is a constant, and *E*_g_ is the bandgap of the semiconductor. Among them, *n* is determined by the optical transition type of a semiconductor. For direct transition, *n* = 1/2, while for the indirect transition, *n* = 2 [31]. According to the calculation result (Fig. 4b), *n* is determined to be 1/2 for the as-formed heterostructures. The bandgap energies (*E*_g_) of Bi_2_WO_6_ and Ag_2_S are estimated to be 3.08 and 1.60 eV, respectively. The bandgap of the as-prepared Bi_2_WO_6_/Ag_2_S heterostructures is estimated to be 2.68 eV, which lies between the bandgaps of Bi_2_WO_6_ and Ag_2_S.

To further explore the bandgap configuration of the as-prepared Bi_2_WO_6_/Ag_2_S heterostructure, values of the relative conduction band (CB) and valence band (VB) position for Bi_2_WO_6_, Ag_2_S, and Bi_2_WO_6_/Ag_2_S are calculated according to the following equations [40, 41]:

$$ {E}_{\mathrm{CB}}=X+{E}_0-0.5{E}_{\mathrm{g}} $$

and

$$ {E}_{\mathrm{VB}}={E}_{\mathrm{CB}}+{E}_{\mathrm{g}} $$

where *E*_g_, *E*_CB_, *E*_VB_, and *X* represent the bandgap energy, conduction band potential, valence band potential, and the electronegativity (the geometric average of the absolute electronegativity of the constituent atoms) of the semiconductor, respectively. *E*_0_ is the scale factor relating to the reference electrode redox level to the absolute vacuum scale (*E*_0_ = −4.5 eV for normal hydrogen electrode). The calculated CB and VB potential of Bi_2_WO_6_, Ag_2_S, and Bi_2_WO_6_/Ag_2_S is summarized in Table 1. Both the *E*_CB_ (−0.27 eV) and *E*_VB_ (1.33 eV) of Ag_2_S are lower than the corresponding *E*_CB_ (0.36 eV) and *E*_VB_ (3.44 eV) values of Bi_2_WO_6_, corresponding to the type-II bandgap alignment when forming the heterostructure. This kind of bandgap alignment will favor for the separation of electrons and holes during the photocatalytic process. Therefore, the Ag_2_S-modified Bi_2_WO_6_ could be excited to produce more electron-hole pairs under the same visible-light illumination, which would result in higher photocatalytic activity.

**Table 1:** Calculated values of bandgap, conduction band (CB) and valence band (VB) of samples Bi_2_WO_6_, Ag_2_S, and Bi_2_WO_6_/Ag_2_S

Sample	Bandgap (eV)	Conduction band (eV)	Valence band (eV)
Bi_2_WO_6_	3.08	0.36	3.44
Ag_2_S	1.60	−0.27	1.33
Bi_2_WO_6_/Ag_2_S	2.68	0.36 (Bi_2_WO_6_)	3.44 (Bi_2_WO_6_)
		−0.27 (Ag_2_S)	1.33 (Ag_2_S)

### Photocatalytic Performance of the Bi_2_WO_6_/Ag_2_S Heterostructure

**Fig. 5:**
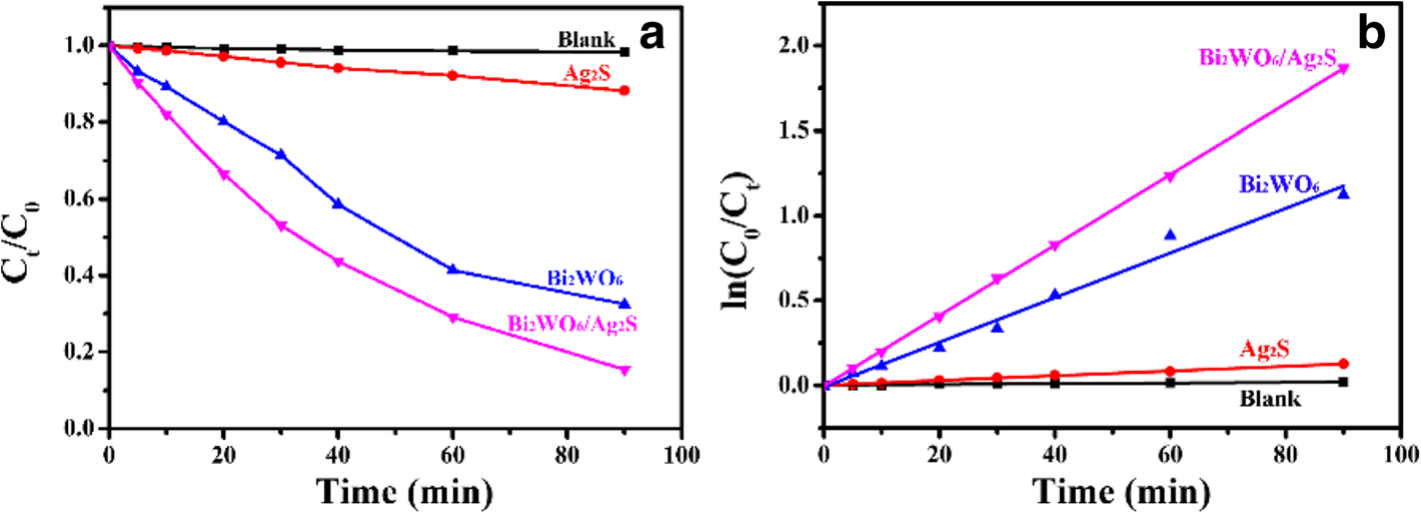
**a** Photocatalytic performances in the presence of different photocatalysts at different time intervals. **b** Plot of ln(*C*_0_/*C*) as a function of irradiation time for photocatalysis of Rh B solution containing the presence of Bi_2_WO_6_, Ag_2_S nanoparticles, Bi_2_WO_6_/Ag_2_S heterostructures under visible-light irradiation, and the blank experiments

To evaluate the photocatalytic activity of the as-prepared Bi_2_WO_6_/Ag_2_S, photodegradation experiments were carried out using Rh B as the target pollutant under visible-light irradiation (*λ* > 400 nm). For comparison purpose, the blank experiments (without photocatalyst) and the photocatalytic activities of bare Bi_2_WO_6_ and Ag_2_S nanoparticles were also investigated. The photocatalytic efficiencies of the samples mentioned above are shown in Fig. 5a. When no photocatalyst is added in the system, the Rh B molecules are very stable under visible-light irradiation and the photodecomposition of Rh B is negligible. When Bi_2_WO_6_ and Ag_2_S are employed, about 68 and 12 % of the Rh B molecules can be decomposed after irradiation for 1.5 h. Although Ag_2_S can absorb light in the whole visible-light region, the photocatalytic activity of Ag_2_S is still lower than Bi_2_WO_6_. The poor photocatalytic activity of Ag_2_S nanoparticles may result from its poor crystallinity, which has been confirmed from the corresponding XRD pattern (Additional file 1: Figure S4). After surface modification with Ag_2_S, the as-formed Bi_2_WO_6_/Ag_2_S heterostructure displays enhanced photocatalytic activity. About 85 % of the Rh B molecules can be decomposed after irradiation with the visible light for 1.5 h. Compared with pure Bi_2_WO_6_ and Ag_2_S, the photocatalytic activity is greatly enhanced, indicating the coupling of Ag_2_S with Bi_2_WO_6_ is an effective way to improve the photocatalytic activities of the photocatalysts.

The kinetic behavior of the degradation process can be described using a pseudo-first-order kinetic model [42]. Therefore, the Langmuir-Hinshelwood model is selected when the concentration of Rh B is in the millimolar range, which is described by the equation

$$ - \ln \frac{C_{\mathrm{t}}}{C_0}=kt+a $$

where *k* is the reaction rate constant, *C*_0_ is the initial concentration of Rh B, and *C*_t_ is the concentration of Rh B at desired reaction time *t*. The plots of −ln(*C*_t_/*C*_0_) versus irradiation time are shown in Fig. 5b. By linear fitting of these plots, the apparent rate constant *k* for Bi_2_WO_6_/Ag_2_S, Bi_2_WO_6_, and Ag_2_S nanoparticles are determined to be 0.02079, 0.01318, and 0.00139 min^−1^, respectively. The corresponding values for all the samples are summarized in Table 2. The photodegradation rate for the as-formed Bi_2_WO_6_/Ag_2_S heterostructure is 1.57 and 14.95 times that of Bi_2_WO_6_ and Ag_2_S nanoparticles, respectively. The enhanced photocatalytic activity of the as-formed Bi_2_WO_6_/Ag_2_S heterostructure can be attributed to the effective electron-hole separation at the interfaces of the two semiconductors.

**Table 2:** Photocatalytic efficiency and rate constant of the Rh B decomposition process in the presence of Bi_2_WO_6_, Ag_2_S, and Bi_2_WO_6_/Ag_2_S

Sample	Photocatalytic efficiency (%)	Rate constant (min^−1^)
Bi_2_WO_6_	67.5	1.318 × 10^−2^
Ag_2_S	11.8	1.390 × 10^−3^
Blank	1.7	1.860 × 10^−4^
Bi_2_WO_6_/Ag_2_S	84.6	2.079 × 10^−2^

**Fig. 6:**
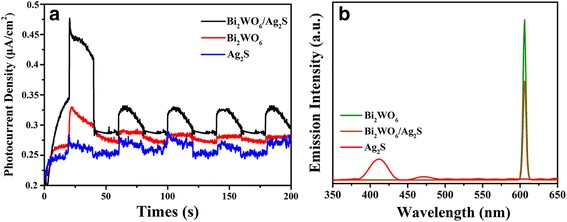
**a** Transient photocurrent response for samples Bi_2_WO_6_, Bi_2_WO_6_/Ag_2_S, and Ag_2_S. **b** PL spectra of samples Bi_2_WO_6_, Bi_2_WO_6_/Ag_2_S (*λ*_Ex_ = 300 nm), and Ag_2_S (*λ*_Ex_ = 200 nm)

To verify our view, both photocurrent and photoluminescence spectra of the as-prepared samples are investigated. Actually, larger magnitude of photocurrent suggests higher charge collection efficiency of the electrode surface, indicating higher separation efficiency of electron-hole pairs [43, 44]. Bi_2_WO_6_/Ag_2_S heterostructures exhibit higher transient photocurrent density than bare Bi_2_WO_6_ and Ag_2_S nanoparticles (Fig. 6a), indicating the enhanced photo-induced electron and hole separation efficiency. Photoluminescence (PL) spectrum is also an effective tool to explore the recombination rate of charge carriers [45–47]. In general, lower PL intensity means lower recombination rate of the electron-hole pairs under light irradiation [48]. The PL spectra (excited at 300 nm) of Bi_2_WO_6_ and Bi_2_WO_6_/Ag_2_S heterostructure are shown in Fig. 6b. Compared to bare Bi_2_WO_6_, the PL emission intensity of the as-formed Bi_2_WO_6_/Ag_2_S heterostructure is much lower. This suggests that the formation of Bi_2_WO_6_/Ag_2_S heterostructure will greatly enhance the separation of the photogenerated electron-hole pairs, thus reducing the recombination rate. Based on the above analysis, the increased separation efficiency of the charge carrier is the main reason for the enhanced photocatalytic performance of the heterostructures.

**Fig. 7:**
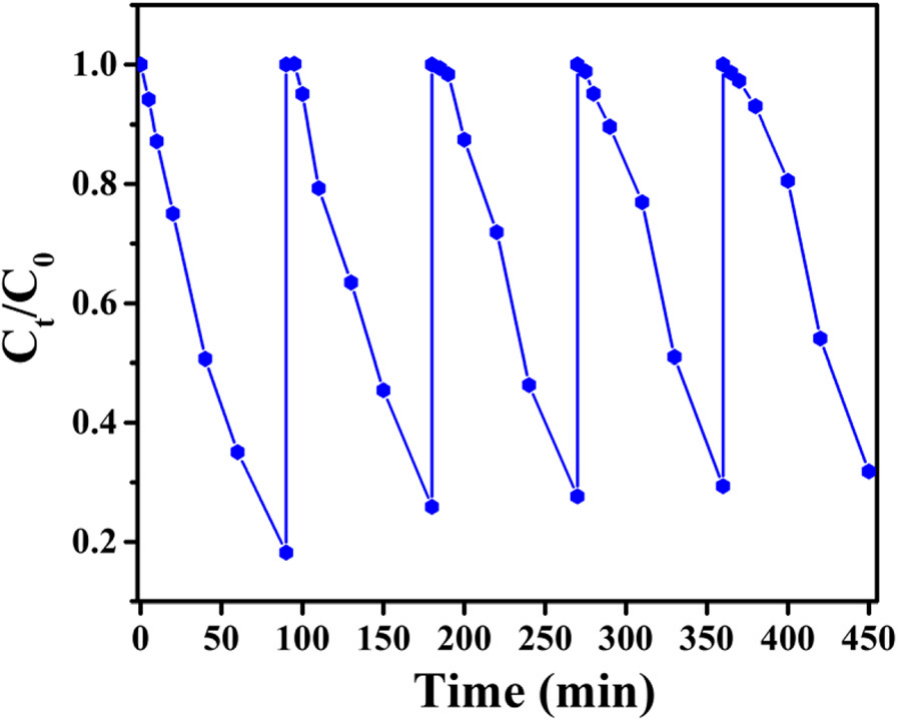
Cycling runs in the photocatalytic degradation of Rh B in the presence of Bi_2_WO_6_/Ag_2_S heterostructures

For the purpose of practical use, the stability of the as-formed Bi_2_WO_6_/Ag_2_S heterostructures was also investigated by the degradation of Rh B under visible-light irradiation (Fig. 7). It should be noted that the as-formed Bi_2_WO_6_/Ag_2_S heterostructure does not exhibit obvious loss in photocatalytic activity even after using for 5 cycles. About 85 % of the initial photocatalytic activity can be retained after being cycled for 5 times. Although the previous reports point that the photocatalysts based on metal sulfides usually suffer from the photocorrosion in the aqueous media containing oxygen, the stability of the as-formed heterostructure is excellent [49, 50]. The excellent stability may result from the formation of the heterostructure and the efficient charge separation at interfaces of the two semiconductors. Considering the excellent photocatalytic activity and good durability, the as-formed Bi_2_WO_6_/Ag_2_S heterostructure is believed to have potential applications in dealing with water contamination.

### Influence of pH on the Photocatalytic Activity of the As-Formed Bi_2_WO_6_/Ag_2_S Heterostructure

To study the influence of pH on the photocatalytic activity of Bi_2_WO_6_/Ag_2_S heterostructure, a series of photocatalytic experiments were carried out under different pH values. In this process, aqueous Rh B solution was adjusted to different initial pH values (2, 3, 4, 5, 6, 7, and 8) using diluted nitrate acid or sodium hydroxide, while keeping other conditions constant. According to the previous reports, the influence of pH mainly takes effect in two aspects. On the one hand, the pH of the solution will influence the adsorption of dye molecules via changing the surface charges of the photocatalysts [51]. The variation in the adsorption of dye molecules will inevitably influence the photocatalytic efficiencies because the photocatalytic process mainly takes place on the surfaces of the photocatalysts. It has been reported that the adsorption of organic pollutants on the surface of the photocatalyst is a prerequisite for efficient photocatalytic degradation because the photocatalytic reaction usually takes place on the surface of the photocatalyst. Usually, strong adsorption benefits the photocatalytic degradation [52, 53]. On the other hand, the pH of the solution also exerts tremendous influence on the molecular structure of dyes, which will determine the attaching modes of Rh B molecules to the surfaces of the photocatalysts [54]. To be specific, the Rh B molecules can attach to the surfaces of the photocatalysts by the carboxylic group or the amino group, and the attaching modes are greatly influenced by the pH of the solution. If Rh B molecules attach to the surfaces of the photocatalysts with the amino group, the photosensitization process will be unfavored. \If Rh B molecules attach to the surfaces of the photocatalysts via the amino group, the photosentization process will be favored, which could be judged by the blue shifts of absorption peaks during the photocatalytic process. So, the influence of pH during the photocatalytic process will be the synergistic effects of the two effects mention above. According to the experimental result, both the photodegradation efficiency and the photodegradation rate of Rh B show monotonous decrease when the pH of the solution increases from 2 to 8 (Fig. 7a, b). About 98 % of the Rh B molecules can be degraded after irradiation for about 90 min when the pH of the solution is 2, whereas about 83 % of the Rh B molecules can be degraded when the pH of the solution is 8. The degradation rate constant is about 0.051 min^−1^ when pH = 2, while the corresponding value decreases to 0.018 min^−1^ when the pH of the solution increases to 8.

**Fig. 8:**
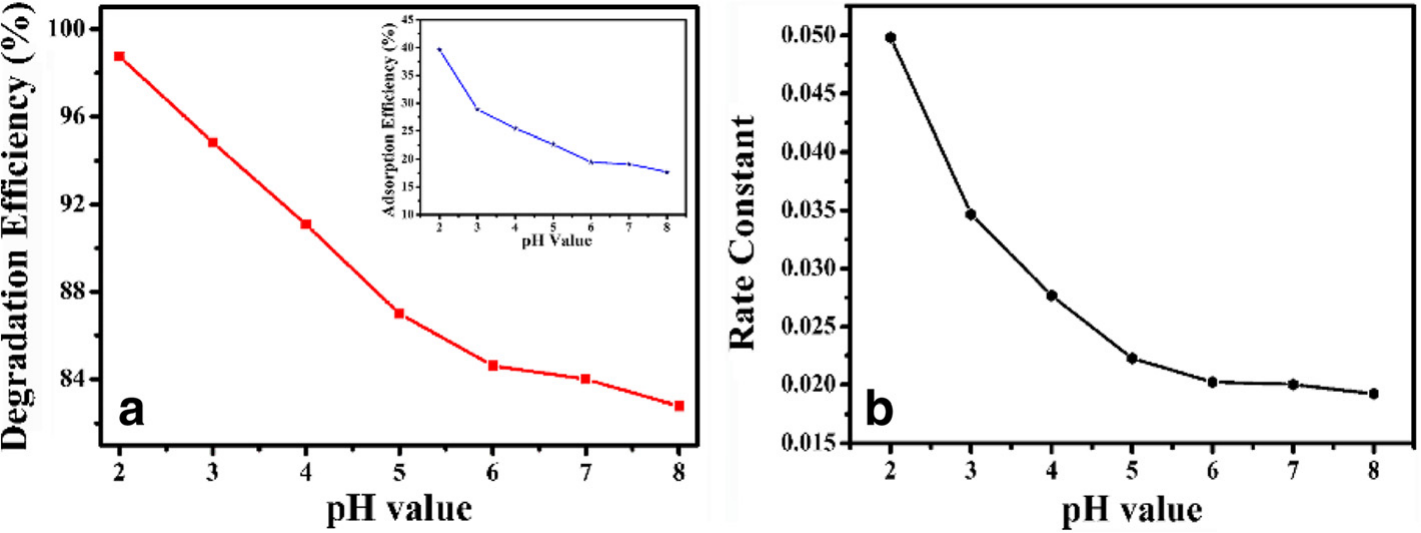
**a** Degradation efficiency and adsorption efficiency (*inset*) at different pH values. **b** The rate constant change of photodegradation for Rh B at different pH values

To disclose the relationship between the pH values and photocatalytic efficiencies in our experiments, the relationship between pH and the adsorption amount of Rh B onto Bi_2_WO_6_/Ag_2_S heterostructure was investigated, and the corresponding result is shown as the inset of Fig. 8a. The experimental results clearly indicate that the adsorption amount of Rh B molecules decreases with the increase of pH from 2 to 8, which is in the same sequence with the changes in photocatalytic efficiencies. To clarify the changes in adsorption amount, zeta potential of the as-prepared Bi_2_WO_6_/Ag_2_S heterostructure was investigated. The result clearly indicates that the point of zero charge is less than 2 (Additional file 1: Figure S5), indicating that the surfaces of Bi_2_WO_6_/Ag_2_S heterostructure are negatively charged in the whole pH region from 2 to 8. Rh B is a cationic dye, which means that an electrostatic repulsion force exists between the Rh B molecules and the photocatalyst. As the pH increases from 2 to 8, the electrostatic repulsion force between the dye molecules and photocatalyst will be strengthened, leading to the decrease in the adsorption amount of the Rh B molecules. Because the photocatalytic process mainly takes place on the surfaces of the photocatalyst, the decrease in the amount of the dye molecules will inevitably lead to the decrease in the photocatalytic activity of the as-obtained heterostructures.

Besides the influence on the adsorption amount of Rh B molecules, the adsorption modes of the Rh B will also be greatly influenced when the pH of the solution changes. According to the previous report, the carboxylic group of will be protonated when the pH is below 3.22 [55] and the carboxylic group will change to its protonated states. Under this situation, the Rh B molecules mainly attached to the surfaces of Bi_2_WO_6_/Ag_2_S heterostructures via the carboxylic group. The benzene ring linked to the carboxylic group is twisted against the chromophoric group, making the electron injection through the carboxylic group impossible. And this effect will greatly suppress the photosensitization process. This hypothesis can be verified by the corresponding spectra of Rh B during the photocatalytic process when the pH of the solution is 2 (Additional file 1: Figure S6a). The blue shift of the absorption band is about 9 nm when the pH of the solution is 2, indicating that the photosensitization process is suppressed. When the pH increased, the Rh B molecules will attach to the surfaces of the heterostructure via the amino groups, which will enable the injection of electrons from Rh B molecules to the photocatalyst. And this kind of attaching mode will favor for the photosensitization process, which has been verified by the spectra of Rh B during the photocatalytic process under different pH values. As it is shown in Additional file 1: Figure S6b–g, the blue shifts of Rh B increase from 13 to 22 nm when the pH of the solution increases from 3 to 8, indicating that the photosensitization process is favored under high pH values.

### Mechanism of the Photocatalytic Process

**Fig. 9:**
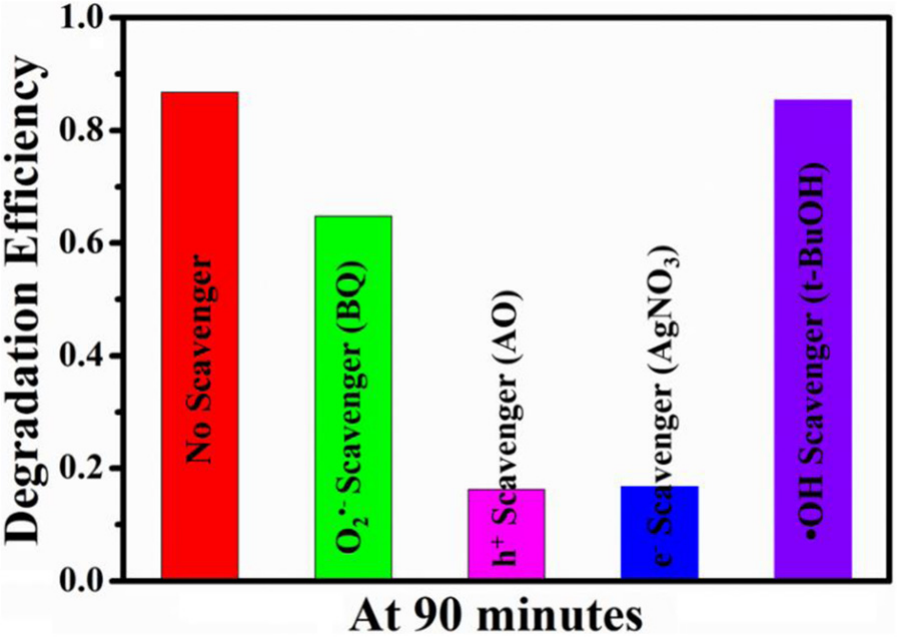
Effects of a series of scavengers on the photodegradation efficiency of Rh B using the as-formed Bi_2_WO_6_/Ag_2_S heterostructure

To investigate mechanism of the photocatalytic process, the trapping experiments were conducted to detect the active species during the photodegradation process of Rh B. The experiments involve the utilization of benzoquinone (BQ), ammonium oxalate (AO), AgNO_3_, and t-BuOH as the scavengers for superoxide radicals (O_2_^·−^), holes (h^+^), electron (*e*^−^), and hydroxyl radicals (·OH), respectively [56]. Before the addition of the photocatalyst, these quenchers should be introduced to the Rh B solution and their amount was 10 mM except for the BQ, which was 1 mM to avoid the reaction with Rh B. After irradiation for 90 min, the corresponding photocatalytic activities are shown in Fig. 9. The photocatalytic activity of Bi_2_WO_6_/Ag_2_S was nearly unchanged when t-BuOH (quencher of ·OH) was added, indicating that hydroxyl radicals are not the driving force for the degradation of Rh B. Oppositely, the photodegradation of Rh B was greatly suppressed by the addition of AO, indicating that the photogenerated holes (h^+^) play vital roles in the photodegradation of Rh B over Bi_2_WO_6_/Ag_2_S heterostructures under the visible-light irradiation. Upon the addition of AgNO_3_ and BQ, the photodegradation efficiencies will also decrease, indicating that both electron (*e*^−^) and superoxide radical anions (O_2_^·−^) are present in the reaction system. Based on the experimental results, the photodegradation process of Rh B in the presence of Bi_2_WO_6_/Ag_2_S heterostructure can be described as follows:

$$ \begin{array}{l}{\mathrm{Ag}}_2\mathrm{S}+\mathrm{h}\mathrm{v}\to {\mathrm{Ag}}_2\mathrm{S}\left({e}_{\mathrm{CB}}^{-}......{\mathrm{h}}_{\mathrm{VB}}^{+}\right)\\ {}{\mathrm{Ag}}_2\mathrm{S}\left({e}_{\mathrm{CB}}^{-}\right)+{\mathrm{Bi}}_2{\mathrm{WO}}_6\to {\mathrm{Ag}}_2\mathrm{S}+{\mathrm{Bi}}_2{\mathrm{WO}}_6\left({e}_{\mathrm{CB}}^{-}\right)\\ {}{\mathrm{Bi}}_2{\mathrm{WO}}_6\left({\mathrm{h}}_{\mathrm{VB}}^{+}\right)+{\mathrm{Ag}}_2\mathrm{S}\to {\mathrm{Bi}}_2{\mathrm{WO}}_6+{\mathrm{Ag}}_2\mathrm{S}\left({\mathrm{h}}_{\mathrm{VB}}^{+}\right)\\ {}{\mathrm{Ag}}_2\mathrm{S}\left({\mathrm{h}}_{\mathrm{VB}}^{+}\right)+\mathrm{R}\mathrm{h}\mathrm{B}\to {\mathrm{CO}}_2+{\mathrm{H}}_2\mathrm{O}\end{array} $$

where *e*^−^_CB_ and h^+^_VB_ represent the electrons in the conduction band and holes in the valence band, respectively.

**Scheme 1:**
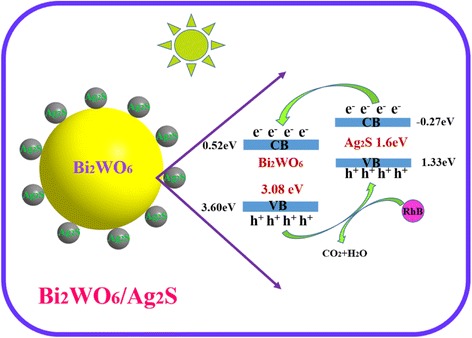
Schematic diagram showing separation of photogenerated charge carriers in sample Bi_2_WO_6_/Ag_2_S

Accordingly, the schematic diagrams for the photocatalytic process in the presence of Bi_2_WO_6_/Ag_2_S heterostructure are presented in Scheme 1. Upon irradiation with the visible light, both Bi_2_WO_6_ and Ag_2_S can be excited. Driven by the built-in electric field, the photogenerated electrons and holes can be separated at the interfaces of the as-formed Bi_2_WO_6_/Ag_2_S heterostructures. Therefore, the electrons and holes can be efficiently separated, offering enough holes for the degradation of dye molecules. As a result, the photocatalytic activity of the as-formed heterostructures was greatly enhanced.

## Conclusions

In this paper, a novel photocatalyst based on Bi_2_WO_6_/Ag_2_S heterostructures was prepared by a facile surface functionalization method. The as-prepared Bi_2_WO_6_/Ag_2_S heterostructure displays enhanced photocatalytic activity for the degradation of Rh B under visible-light irradiation compared to its individual components. The decomposition rate for Rh B in the presence of Bi_2_WO_6_/Ag_2_S is about 1.6 times higher than the corresponding value when Bi_2_WO_6_ is used as the photocatalyst. The PL and photocurrent measurement were applied to verify the effective separation of electron-hole pairs, which indicate that the enhanced separation of the charge carriers is the main reason for the enhanced photocatalytic activity of the heterostructure. The pH of the solution exerts tremendous influence on the photocatalytic activity of the as-formed heterostructures. According to the active-species-trapping experiments, the photogenerated holes (h^+^) are determined to be the main reactive species for Bi_2_WO_6_/Ag_2_S in this photocatalytic process.

## Additional file

Additional file 1: Figures S1–S6. The EDS, BET surface area, and Zeta potential analysis for the as-formed heterostructures, the XRD pattern of Ag_2_S, and the temporal evolution of Rh B absorption spectra over Bi_2_WO_6_/Ag_2_S heterostructure at different pH values. Figure S1. Elemental mapping and EDX spectra of the Bi_2_WO_6_/Ag_2_S heterostructure. Figure S2. EDS spectra of the composite photocatalysts Bi_2_WO_6_/Ag_2_S. Figure S3. Nitrogen adsorption-desorption isotherms and the pore size distribution curve (inset) of sample (a) Bi_2_WO_6_ and (b) Bi_2_WO_6_/Ag_2_S. Figure S4. XRD pattern of Ag_2_S. Figure S5. Zeta potential for a suspension containing 1 g L of sample Bi_2_WO_6_/Ag_2_S in the presence of KCl (10^−3^ M) at different pH values. Figure S6. The temporal evolution of Rh B absorption spectra over Bi_2_WO_6_/Ag_2_S heterostructure at different pH values.
