# Supplementary material for: Facile Fabrication of Bi2WO6/Ag2S Heterostructure with Enhanced Visible-Light-Driven Photocatalytic Performances
Source: Nanoscale Res Lett. 2016 Mar 8;11:126. doi: 10.1186/s11671-016-1319-7 (PMC4781791; doi:10.1186/s11671-016-1319-7)
Supplement: Additional file 1: Figures S1–S6. — The EDS, BET surface area, and Zeta potential analysis for the as-formed heterostructures, the XRD pattern of Ag2S, and the temporal evolution of Rh B absorption spectra over Bi2WO6/Ag2S heterostructure at different pH values. Figure S1. Elemental mapping and EDX spectra of the Bi2WO6/Ag2S heterostructure. Figure S2. EDS spectra of the composite photocatalysts Bi2WO6/Ag2S. Figure S3. Nitrogen adsorption-desorption isotherms and the pore size distribution curve (inset) of sample (a) Bi2WO6 and (b) Bi2WO6/Ag2S. Figure S4. XRD pattern of Ag2S. Figure S5. Zeta potential for a suspension containing 1 g L of sample Bi2WO6/Ag2S in the presence of KCl (10−3 M) at different pH values. Figure S6. The temporal evolution of Rh B absorption spectra over Bi2WO6/Ag2S heterostructure at different pH values. [file 11671_2016_1319_MOESM1_ESM.docx]

**Facile fabrication of Bi_2_WO_6_/Ag_2_S heterostructure with Enhanced Visible-Light-Driven Photocatalytic performances**

Rongfeng Tang, Huaifen Su, Yuanwei Sun, Xianxi Zhang, Lei Li, Caihua Liu, Suyuan Zeng *and Dezhi Sun *

Shandong Provincial Key Laboratory of Chemical Energy Storage and Novel Cell Technology, Department of Chemistry and Chemical Engineering, Liaocheng University, Liaocheng 252059, China

Corresponding author: Suyuan Zeng and Dezhi Sun. Tel: +86-635-8230614, Fax: +86-635-8230196. Email: drzengsy@163.com, sundezhi@lcu.edu.cn

**Additional file 1**


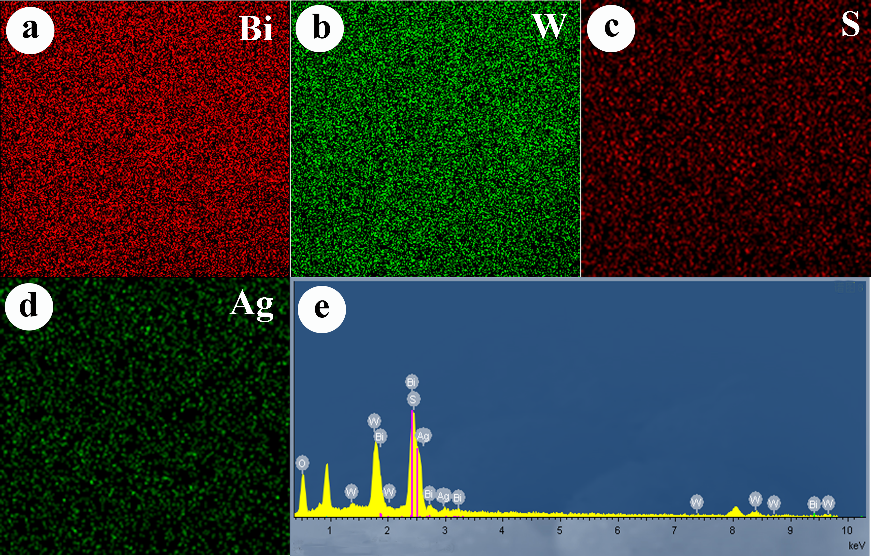


**Figure S1.** (a-d) Elemental mapping, (e) EDX spectra of the Bi_2_WO_6_/Ag_2_S heterostructure.


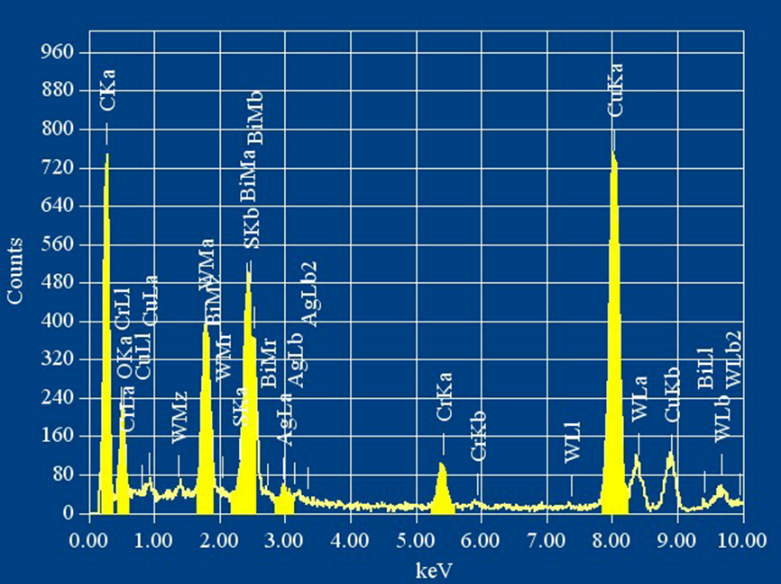


**Figure S2.** EDS spectra of the composite photocatalysts Bi_2_WO_6_/Ag_2_S


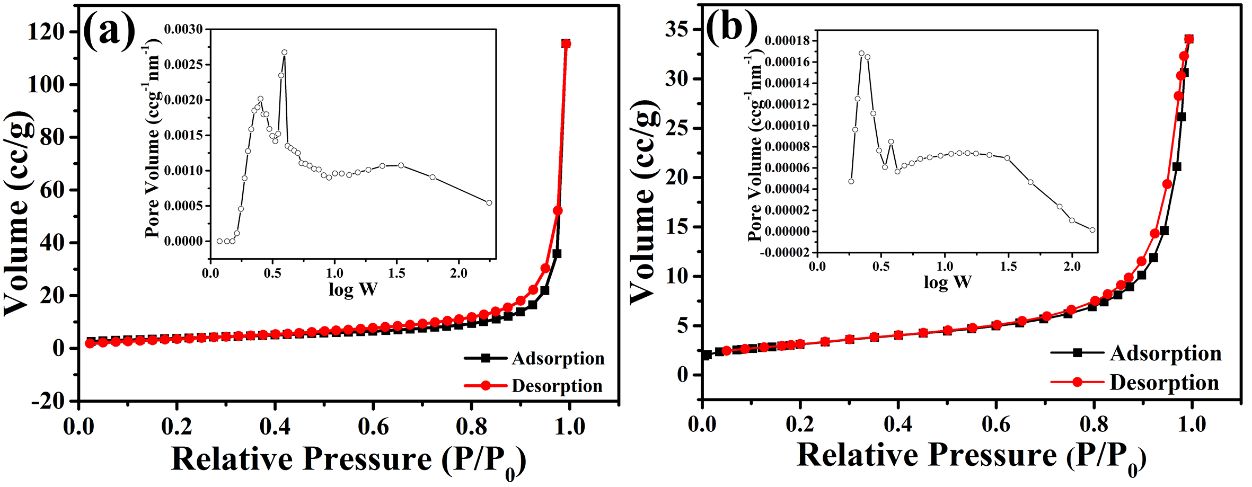


**Figure S3.** Nitrogen adsorption-desorption isotherms and the pore size distribution curve (inset) of sample (a) Bi_2_WO_6_ and (b) Bi_2_WO_6_/Ag_2_S.

*

*

**Figure S4.** XRD pattern of Ag_2_S.





**Figure S5.** Zeta potential for a suspension containing 1 g/L of sample Bi_2_WO_6_/Ag_2_S in the presence of KCl (10^-3^ M) at different pH value.


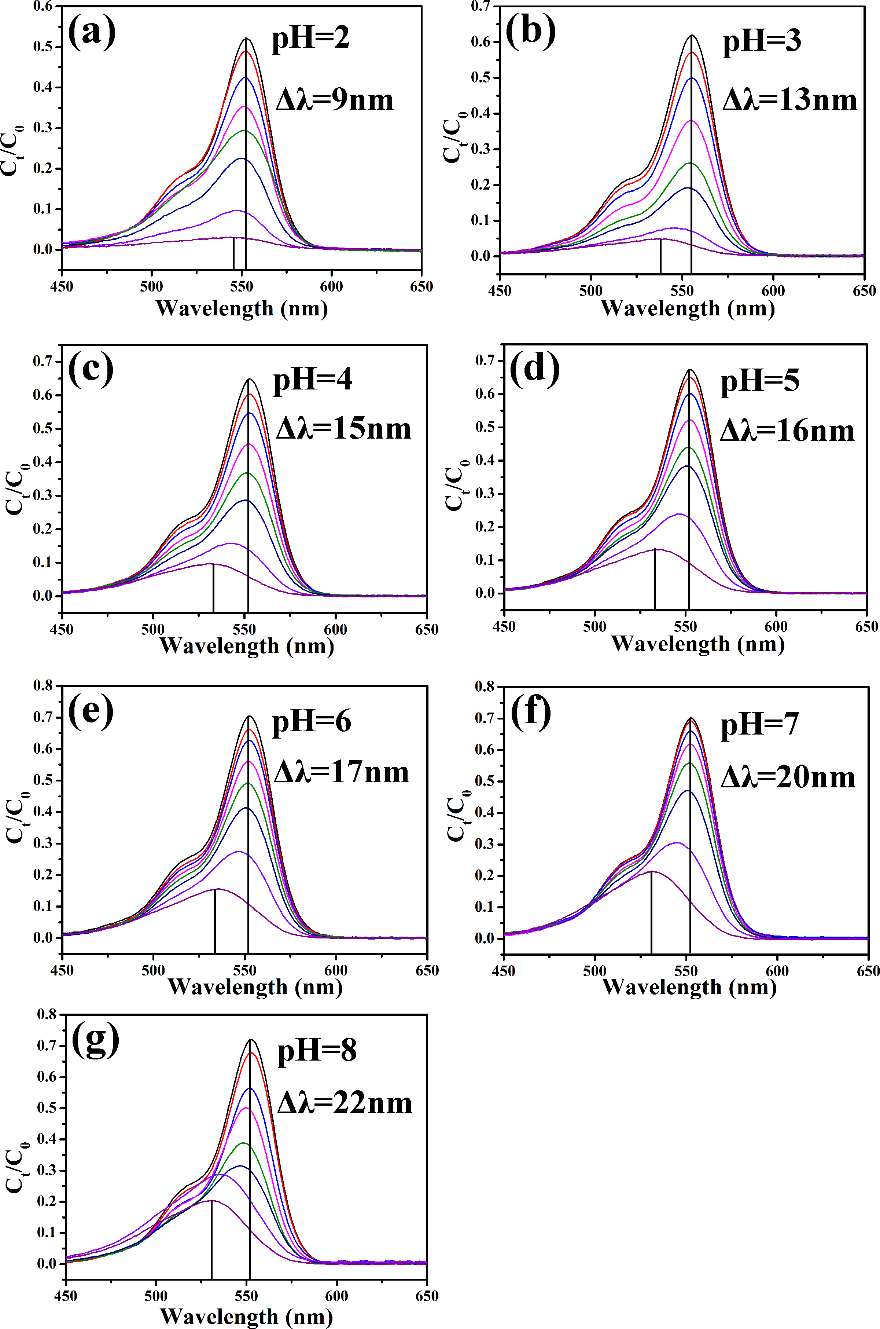


**Figure S6.** The temporal evolution of RhB absorption spectra over Bi_2_WO_6_/Ag_2_S heterostructure at different pH value.
